# Esophageal Stent for Refractory Variceal Bleeding: A Systemic Review and Meta-Analysis

**DOI:** 10.1155/2016/4054513

**Published:** 2016-07-19

**Authors:** Xiao-Dong Shao, Xing-Shun Qi, Xiao-Zhong Guo

**Affiliations:** Department of Gastroenterology, General Hospital of Shenyang Military Area Command, Shenyang 110016, China

## Abstract

*Background*. Preliminary studies suggest that covered self-expandable metal stents may be helpful in controlling esophageal variceal bleeding.* Aims*. To evaluate the effectiveness and safety of esophageal stent in refractory variceal bleeding in a systematic review and meta-analysis.* Methods*. A comprehensive literature search was conducted on PubMed, EMBASE, and Cochrane Library covering the period from January 1970 to December 2015. Data were selected and abstracted from eligible studies and were pooled using a random-effects model. Heterogeneity was assessed using *I* ^2^ test.* Results*. Five studies involving 80 patients were included in the analysis. The age of patients ranged from 18 to 91 years. The mean duration of follow-up was 46.8 d (range, 30–60 d). The success rate of stent deployment was 96.7% (95% CI: 91.6%–99.5%) and complete response to esophageal stenting was in 93.9% (95% CI: 82.2%–99.6%). The incidence of rebleeding was 13.2% (95% CI: 1.8%–32.8%) and the overall mortality was 34.5% (95% CI: 24.8%–44.8%). Most of patients (87.4%) died from hepatic or multiple organ failure, and only 12.6% of patients died from uncontrolled bleeding. There was no stent-related complication reported and the incidence of stent migration was 21.6% (95% CI: 4.7%–46.1%).* Conclusion*. Esophageal stent may be considered in patients with variceal bleeding refractory to conventional therapy.

## 1. Introduction

Esophageal varices are portosystemic collateral venous channels related to portal hypertension and present in nearly 50% of patients diagnosed with cirrhosis [1]. They initially develop as small varices that gradually dilate at a rate of 5% per year. Acute variceal bleeding is a severe complication of portal hypertension causing 70% of all upper gastrointestinal bleeding episodes in patients with portal hypertension [2]. With the use of current prophylactic therapies of variceal bleeding, including nonselective beta-blockers and band ligation, the rate of first variceal bleeding is about 8% per year [3]. Risk factors of variceal bleeding mainly include the severity of liver disease, the size of varix, and the presence of red wale marks [4]. Hemodynamic studies suggest a close association of hepatic venous pressure gradient with the risk of variceal bleeding [5]. Prognostic factors for death include the severity of variceal bleeding, the degree of hepatic dysfunction, and the development of complications including acute renal failure and bacterial infections [6]. Mortality of patients with variceal bleeding has decreased significantly over the last two decades with the implementation of intensive care management, including the use of antibiotic prophylaxis and endoscopic variceal band ligation [7, 8]. However, the treatment of refractory bleeding and prevention of early rebleeding are still a challenge for physicians [9].

The aim of treatment of acute variceal bleeding is to correct hypovolemia, prevent complications, and achieve hemostasis. After resuscitation, airway protection, and prevention of complication, the initial approach for variceal bleeding is a combination of vasoactive drugs, antibiotics, and endoscopic therapy [10]. About 80%–90% of acute variceal bleeding episodes are successfully controlled by endoscopic therapy [11]. In 10%–20% of patients acute variceal bleeding is not controlled with this primary endoscopic and pharmacological therapy, which is known as refractory variceal bleeding [12]. More aggressive therapies may be used to deal with refractory variceal bleeding. Early TIPS should be considered in patients at high risk of treatment failure after initial endoscopic and pharmacological therapy [13]. Although rescue TIPS is very effective in controlling the bleeding, the mortality is high (25–60%) due to the poor condition of patients [14]. Balloon tamponade aims at achieving hemostasis by direct compression of the bleeding varices. However, after deflating the balloon, the recurrence rate of bleeding is about 50% [15]. Complications can occur in more than 25% of the patients treated with balloon tamponade with fatal ones in 5% of the cases. So it is recommended that balloon tamponade should only be used by skilled and experienced personnel in intensive care facilities [16]. Additionally, the use of the tube is highly unpleasant for patients. Surgical procedures are employed less frequently with the development of endoscopic therapy and TIPS. Although surgical procedures can effectively control variceal bleeding, the mortality remains high (45–75%) and hepatic encephalopathy is a major complication after shunt procedures [17]. The above-mentioned limitations prompt physicians to seek other modalities to deal with refractory esophageal variceal bleeding.

Self-expandable metal stents (SEMSs) are mainly used in various benign and malignant esophageal diseases, such as stricture, tracheoesophageal fistula, perforation, and achalasia [18]. Anecdotal experience suggests that the covered SEMSs may be useful in controlling esophageal variceal bleeding [19–25]. Since refractory variceal bleeding is an uncommon complication of cirrhosis, most of studies on the effectiveness of esophageal stent have been limited to a small number of patients [26–32]. A recent randomized controlled trial included only 13 patients undergoing esophageal stenting for refractory variceal bleeding [33]. The purpose of this study was to evaluate the effectiveness and safety of esophageal stent in patients with refractory variceal bleeding by pooling all available evidence in a systematic review with meta-analysis.

## 2. Materials and Methods

### 2.1. Literature Search

A comprehensive literature search was conducted using PubMed, EMBASE, and Cochrane Library for the period from January 1970 to December 2015. The search terms included, in different combinations, “esophageal stent”, “self-expandable metal stents”, “variceal bleeding”, “variceal hemorrhage”, and “endoscopic hemostasis”. The search was limited to studies in humans published in English. References of eligible articles and review articles were manually searched.

### 2.2. Selection of Articles

The selection criteria were studies in (1) patients with cirrhosis irrespective of etiology; (2) patients with refractory variceal bleeding; and (3) series that included at least 10 patients. Case reports or series with fewer than 10 patients were excluded. After excluding duplicate articles, article titles and abstracts were screened by a reviewer (SXD). Each eligible article was reviewed in full text.

### 2.3. Data Extraction

Data were abstracted by the same reviewer and entered into an Excel spreadsheet (Microsoft Corp., Redmond, Washington). The following information was abstracted from each study: author, country, publication year, publication type, study design, participants, and outcome of interest (success rate of stent deployment, response to esophageal stent, rebleeding rate, overall mortality, cause of death, stent-related complications, and incidence of stent migration).

### 2.4. Definitions

*Refractory Variceal Bleeding*. Patients with active variceal bleeding were unresponsive to pharmacologic and endoscopic therapy and required transfusion.

*Response to Esophageal Stent*. Clinically, response was considered as complete hemostasis if the patients' symptoms of bleeding resolved. No response was defined as persistent or worsening symptoms of bleeding. Endoscopically, response was defined as no active bleeding or oozing from a varix on endoscopy. No response was defined as active bleeding or oozing found on endoscopy. Eligible studies used endoscopic and/or clinical criteria to assess the response to esophageal stent. 

*Esophageal Stent-Related Complications*. Esophageal stent-related complications include esophageal tear or perforation, pulmonary dysfunction, aspiration pneumonia, asphyxia, and esophageal ulcer leading to bleeding which need further specific treatment. 

*Stent Migration*. Esophageal stent migration was defined as stent migrating proximally or distally from the place where stents were deployed initially. Radiological examinations were necessary. 

*Overall Mortality*. Overall mortality was defined as any death events throughout the follow-up period. The follow-up period varied across the studies, with the longest duration being 60 d.

### 2.5. Statistical Analysis

Data from eligible studies were pooled using a random-effects model with StatsDirect statistical software Version 2.7.8. Outcomes are expressed as proportions (percentages) with 95% CIs. The pooled analyses are presented as forest plots. Statistical heterogeneity between studies was assessed using the Cochran *Q* test and *I* ^2^ statistic. *I* ^2^ value of greater than 50% or a *P* value of less than 0.05 for the *Q* statistic was taken to indicate significant heterogeneity.

## 3. Results

### 3.1. Literature Search Results

**Figure 1:**
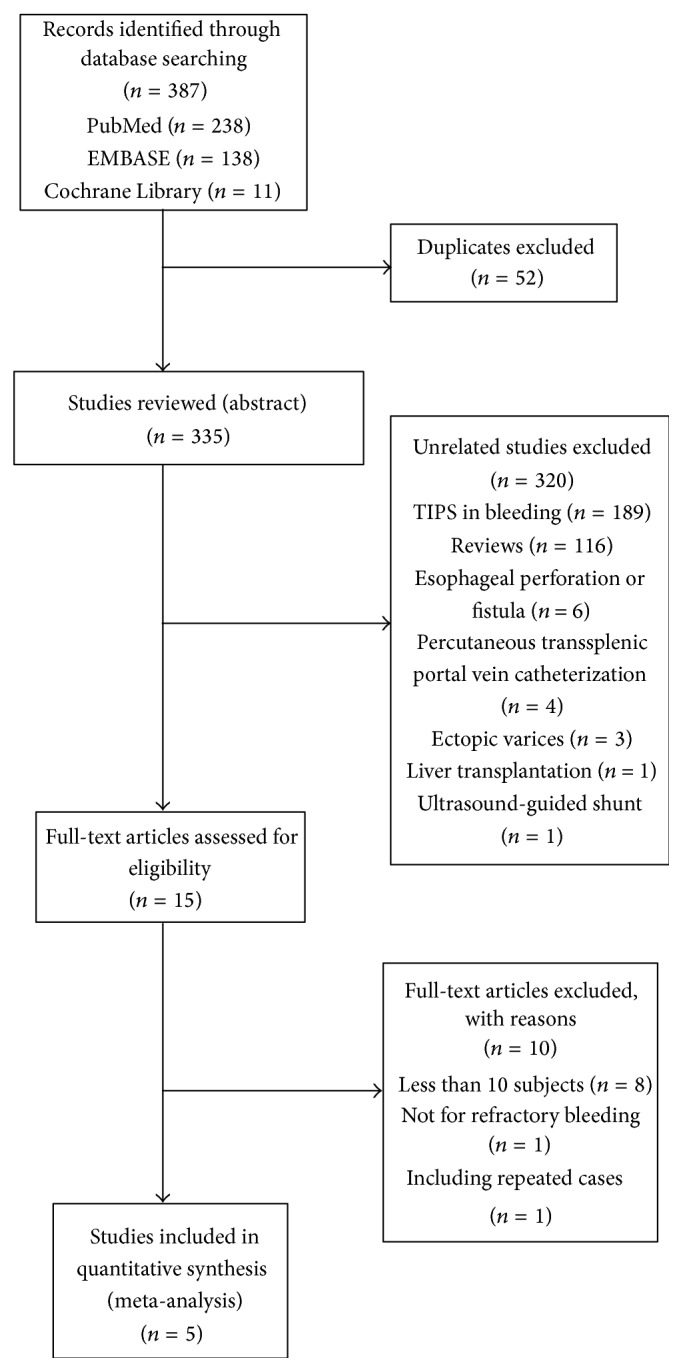
Study selection flow chart. Of a total of 387 studies, only 5 studies met selection criteria. TIPS indicates transjugular intrahepatic portosystemic shunt.

Five studies involving a total of 80 patients were included in the analyses. Eight studies were excluded because each had a small number of study subjects.  Figure 1 summarizes the results of the literature search.  Table 1 summarizes the characteristics of the 5 eligible studies.

### 3.2. Characteristics of Study Participants

Seventy-one patients were male. The age ranged from 18 to 91 years. The mean duration of follow-up was 46.8 d (range, 30–60 d).  Table 2 shows the results of the various outcomes of the individual studies.

### 3.3. Stent Deployment

**Figure 2:**
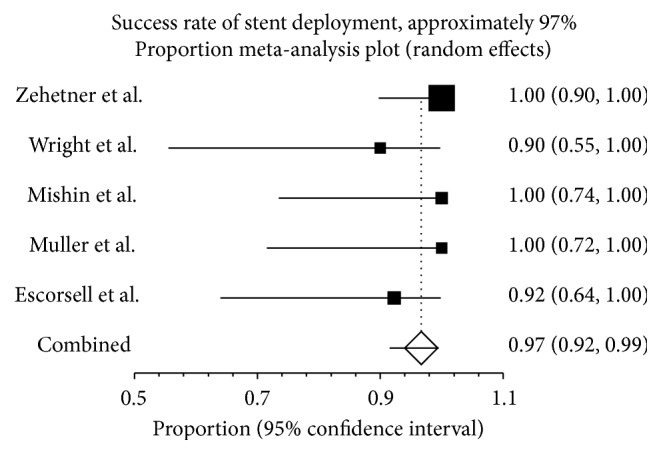
Deployment of esophageal stent in patients with refractory variceal bleeding. The esophageal stents were successfully deployed in 96.7% (95% CI: 91.6%–99.5%) of the 80 patients in the 5 studies. There was no heterogeneity among the studies (*P* = 0.37).

The success rate of stent deployment was 96.7% (95% CI: 91.6%–99.5%) (Figure 2). Heterogeneity was not significant among the studies (*I* ^2^ = 6.8%; *P* = 0.37).

### 3.4. Response to Esophageal Stent

**Figure 3:**
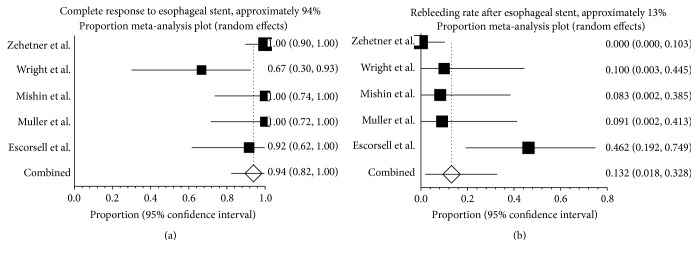
Response to esophageal stent in refractory variceal bleeding. (a) Forest plot shows that 93.9% (95% CI: 82.2%–99.6%) of the 80 patients in the 5 studies had a complete response (resolution of acute variceal bleeding without further need for other treatments) after deployment of esophageal stent. There was evidence of heterogeneity among the studies (*P* = 0.03). (b) Just over one-tenth (13.2%) of the patients treated with esophageal stents rebled after this procedure. There was evidence of heterogeneity among studies (*P* = 0.00).

Complete response to esophageal stenting was in 93.9% (95% CI: 82.2%–99.6%) (Figure 3(a)). Heterogeneity was significant among the studies (*I* ^2^ = 62.5%; *P* = 0.03). The incidence of rebleeding was 13.2% (95% CI: 1.8%–32.8%) (Figure 3(b)). Heterogeneity was significant among the studies (*I* ^2^ = 78.1%; *P* = 0.00).

### 3.5. Mortality

**Figure 4:**
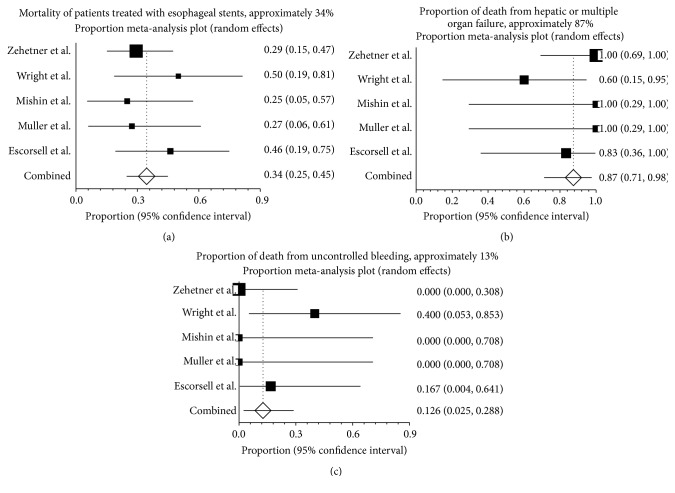
Mortality and causes of death of patients treated with esophageal stents. (a) Forest plot shows that about one-third [34.5% (95% CI: 24.8%–44.8%)] of the 80 patients in the 5 studies died within 30 or 60 d of undergoing esophageal stents. There was no evidence of heterogeneity among studies (*P* = 0.60). (b) About nine-tenth [87.4% (95% CI: 71.2%–97.5%)] of deaths were due to hepatic or multiple organ failure in patients treated with esophageal stents. There was no evidence of heterogeneity among studies (*P* = 0.25). (c) Just over one-tenth [12.6% (95% CI: 2.5%–28.8%)] of deaths were contributed to uncontrolled bleeding. There was no evidence of heterogeneity among studies (*P* = 0.30).

The overall mortality was 34.5% (95% CI: 24.8%–44.8%) (Figure 4(a)). Heterogeneity was not significant among the studies (*I* ^2^ = 0; *P* = 0.60). 87.4% (95% CI: 71.2%–97.5%) of patients died from hepatic or multiple organ failure (Figure 4(b)). Only 12.6% (95% CI: 2.5%–28.8%) of patients died from uncontrolled bleeding (Figure 4(c)).

### 3.6. Complications

There was no stent-related complication reported.

### 3.7. Stent Migration

**Figure 5:**
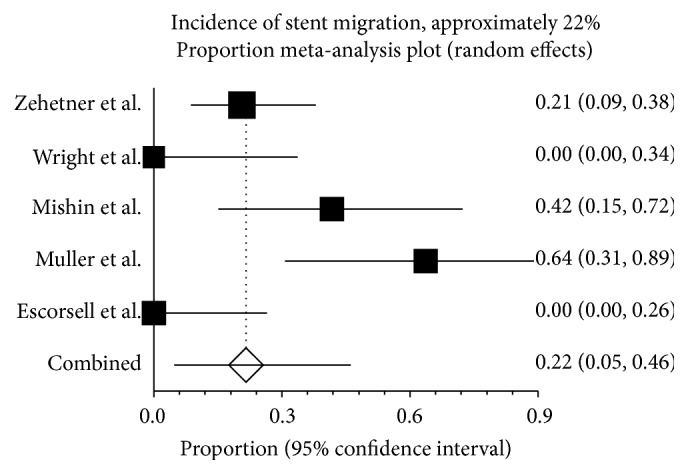
Stent migration after stent deployment. Stent migration was noted in about one-fifth [21.6% (95% CI: 4.7%–46.1%)] of the 80 patients in the 5 studies. There was, however, evidence of heterogeneity among the studies (*P* = 0.00).

The incidence of stent migration was 21.6% (95% CI: 4.7%–46.1%) (Figure 5). Heterogeneity was significant among the studies (*I* ^2^ = 81.6%; *P* = 0.00).

## 4. Discussion

This study shows the following: (1) esophageal stent was successfully deployed in 96.7% of patients with refractory variceal bleeding; (2) after successful deployment, the hemostasis rate was 93.9%; (3) the rate of rebleeding after esophageal stent is 13.2%; (4) no stent-related complications were reported in the 5 studies; (5) the overall mortality of patients was 34.5%; (6) a majority of patients with refractory variceal bleeding died of hepatic or multiple organ failure and only a minority of patients died from uncontrolled bleeding.

Variceal bleeding is a lethal complication of liver cirrhosis. When acute variceal bleeding fails to respond to pharmacological or endoscopic treatment, balloon tamponade is often undertaken [34, 35]. Although balloon tamponade is effective in controlling bleeding, it can be associated with complications such as perforation, asphyxia, and aspiration pneumonia [36–40]. And when balloon is extracted, the rebleeding rate can be as high as 50% [6]. TIPS is an alternative for refractory variceal bleeding and can achieve complete response in most cases [12]. Hepatic encephalopathy is the most common complication of TIPS [41]. Acute and acute-on-chronic liver failure are regarded as contraindications of TIPS [42, 43]. If these patients had undergone TIPS at the time of bleeding, the risk of early death after TIPS would have been very high, approaching 60% [44].

Esophageal stent is a nonsurgical approach that maintains the patency of esophagus in malignant or benign esophageal obstructions [45]. Esophageal stent is also used in dealing with esophageal perforation and fistula with a satisfactory outcome [18]. However, few studies explored its use in patients with refractory variceal bleeding. Consequently, the evidence on the effectiveness of esophageal stent in refractory variceal bleeding has been limited to a small number of study participants. Pooled results from 5 studies found a wide range of response and rebleeding rates, perhaps due to the lack of statistical power. In this study, we combined the data from these small studies, which allowed us to provide the best evidence on the effectiveness of esophageal stent in refractory variceal bleeding.

The stents used in the 5 studies are self-expandable covered esophageal metal stent (SX-ELLA-Danis, Czech Republic) that are specifically produced for controlling variceal bleeding [19]. The success rate of deploying stent in patients with acute variceal bleeding is 96.7%. This proportion is well within the range for success rate of esophageal stenting in patients with malignant and benign esophageal strictures. Serious conditions, such as esophageal perforation [24] and acute bleeding, may not hinder the deployment of esophageal stents. Of course, the specific design of stents used in these studies may contribute to such a high success rate of procedures. However, in the earliest report of this procedure, five standard esophageal stents were also successfully inserted into expected positions with radiological guide [19]. Some reports indicated that the deployment of esophageal stent in patients with acute variceal bleeding is not a difficult procedure and specific designs of stents are more adequate in emergency room or intensive care unit without radiological facility [24]. In rare cases, the stents were inserted blindly without either X-ray or endoscopy [24]. However, Müller et al. pointed out that adequate training and exercise are necessary for successful deployment of esophageal stent in patients with acute variceal bleeding and blind stent implantation should not be advocated with concern of adverse events [26].

Refractory variceal bleeding is controlled after stent deployment in 93.9% of patients. Balloon tamponade is widely used in variceal bleeding which is adopted in 17.4% of patients with rebleeding [46]. Reportedly, 80%–90% of patients with refractory variceal bleeding treated with balloon tamponade achieved hemostasis [15]. This pooled analysis shows that in general the efficacy of esophageal stent on controlling bleeding seems not to be inferior to balloon tamponade. In the only RCT comparing esophageal stent and balloon tamponade, the primary composite endpoint (absence of digestive bleeding with absence of serious adverse events and survival at day 15) was achieved in 66% of the cases in the esophageal stent group but only in 20% in the balloon tamponade group [33]. According to this RCT, esophageal stent appears to be superior to balloon tamponade in treating refractory bleeding. In addition, esophageal stent allows patients to take food and drugs orally and undergo necessary diagnostic procedures including endoscopy. Esophageal stent is more comfortable than balloon tamponade for patients. The endotracheal intubation is not necessary for esophageal stenting, but balloon tamponade usually requires airway protection with intubation [38]. Balloon tamponade could be maintained for a maximum of 24–48 h to avoid esophageal or gastric pressure necrosis, but esophageal stents could remain in place for a longer period (range: 0–24 d). Thus, in hospitals where TIPS is not available, the longer retention time of stent is critical for safe transfer of patients to other medical centers. However, gastric varices will not be compressed by esophageal stent, so balloon tamponade still has a role in the management of gastric variceal bleeding.

With the use of banding ligation, vasoactive drugs, and antibiotics, the mortality of patients with variceal bleeding has been improved in recent years [47–50]. But initial failure to control bleeding or early rebleeding is usually associated with higher mortality [51]. The mortality rate increases to 80% if the initial treatment fails to stop acute bleeding [19]. In our study, the hemostasis rate was 94%, but the overall mortality is still 34%. These figures are higher than a general 6-week mortality rate of 15–20% using standard techniques [8]. This high mortality is attributed to the fact that all studies were conducted in high-risk patients with severe underlying liver diseases. The patients who are not responsive to standard therapy usually have advanced diseases and worse liver function. Child-Pugh class is the main predictor of outcome in patients with variceal bleeding [52]. Three of the 5 studies clearly recorded the Child-Pugh score, in which the proportion of patients with Child classes B and C is 77%–100%. Indeed, most of death cases (87%) were from hepatic or multiple organ failure. The mortality rate of 34% in our study is lower than the results of previous studies conducted in patients with refractory variceal bleeding treated with other modalities [53].

The safety of esophageal stent in patients with variceal bleeding should be carefully assessed. The design of SX-ELLA-Danis stent with a protective pressure valve decreases the risk of perforation due to overinflated gastric balloon in esophagus [19]. In fact, two patients with concurrent variceal bleeding and esophagus perforation were successfully treated with this specifically designed stent [24]. Esophageal stenting followed by TIPS may be a reasonable choice for patients with variceal bleeding and perforation induced by balloon tamponade. There is no stent-related complication reported in the 5 studies, but an acute deterioration of pulmonary function was reported in a case report [23]. So the safety of this procedure should be further investigated in a larger number of patients. Stent migration occurred in about one-fifth of patients but the dislocation, proximal or distal, is not associated with any major damage such as rebleeding, perforation, or obstruction. With close monitoring the stent migration was easily detected and repositioned with endoscopy.

Collectively, esophageal stent may be considered as an alternative to refractory variceal bleeding in future. However, our conclusions should be cautiously interpreted due to the following limitations. First, there is significant heterogeneity among studies in terms of length of follow-up and important endpoints such as complete response, incidence of rebleeding, and stent migration. Second, the quality of the studies is mediocre. Only one study was a small RCT. Third, the characteristics of the patients included in the studies are reported incompletely. Two studies did not report the Child-Pugh classification. Two studies did not report the presence of HCC. Two studies did not report the presence of previous bleeds. Fourth, the overall mortality in these studies is disappointingly high. Fifth, the stent has to be removed after a period of 1-2 weeks. Thus, additional strategies to lower portal pressure are warranted. The best combination of different modalities with esophageal stent needs further studies. In all of the 5 studies included in this analysis, esophageal stents serve as a temporary or bridging treatment followed by other therapies.

## Figures and Tables

**Table 1:** Study characteristics.

Author	Country	Publication year	Publication type	Study design	Number of cases	Gender (M/F)	Age (years)	Child class A (%)	Child classes B + C (%)	HCC (%)	Previous bleeding episode (%)	Previous BT (%)
Zehetner et al. [20]	Austria	2008	Full text	Retrospective	34	33/1	Mean: 56 (32–91)	0 (0%)	34 (100%)	NA	24 (70.59%)	6
Wright et al. [24]	UK	2010	Full text	Retrospective	10	9/1	Mean: 49 (18–70)	NA	NA	2 (20%)	NA	5
Mishin et al. [32]	Moldova	2013	Abstract	Retrospective	12	8/4	Mean: 46.92 (24–62)	NA	NA	NA	NA	NA
Müller et al. [26]	Germany	2015	Full text	Retrospective	11	8/3	Mean: 64 (43–72)	1 (9.09%)	10 (90.91%)	3 (27.27%)	5 (45.45%)	NA
Escorsell et al. [33]	Spain	2016	Full text	RCT	13	13/0	Median: 69 (40–81)	3 (23.08%)	10 (76.92%)	2 (15.38%)	6 (46.15%)	0

**Table 2:** Outcomes of esophageal stent.

Author	Success of deployment	Time of stenting (d)	Stent migration	Stent-related complications	Bleeding controlled	TIPS after stent	Follow-up (d)	Rebleeding	30 d mortality	42 d mortality	60 d mortality	Death from uncontrolled bleeding	Death from hepatic or multiple organ failure
Zehetner et al. [20]	34 (100%)	1–14	7	0	34 (100%)	8	60	0 (0%)	9 (26.47%)	NA	10 (29.41%)	0	10 (100%)
Wright et al. [24]	9 (90%)	6–14	0	0	6 (66.67%)	1	60	1 (11.11%)	NA	5 (50%)	NA	2 (40%)	3 (60%)
Mishin et al. [32]	12 (100%)	NA	5	0	12 (100%)	NA	30	1 (8.33%)	3 (25%)	NA	NA	0	3 (100%)
Müller et al. [26]	11 (100%)	5–24	7	0	11 (100%)	2	42	1 (9.09%)	NA	3 (27.27%)	NA	0	3 (100%)
Escorsell et al. [33]	12 (92.31%)	0–12	0	0	11 (91.67%)	4	42	6 (50%)	NA	6 (50%)	NA	1 (16.67%)	5 (83.33%)
